# Comparative Bioavailability of Vitamin C After Short-Term Consumption of Raw Fruits and Vegetables and Their Juices: A Randomized Crossover Study

**DOI:** 10.3390/nu17213331

**Published:** 2025-10-23

**Authors:** Mijoo Choi, Juha Baek, Jung-Mi Yun, Young-Shick Hong, Eunju Park

**Affiliations:** 1 RISE Bio-Center, Kyungnam University, Changwon 51767, Republic of Korea; mijjoo@kyungnam.ac.kr; 2 Department of Food and Nutrition, Kyungnam University, Changwon 51767, Republic of Korea; kyungsoo0318@naver.com; 3 Department of Food and Nutrition, Chonnam National University, Gwangju 61186, Republic of Korea; sosung75@jnu.ac.kr

**Keywords:** ascorbic acid, fruit and vegetable juices, biological availability, absorption

## Abstract

Background/Objectives: Vitamin C plays a vital role in human health, functioning as a powerful antioxidant and enzymatic cofactor. Although vitamin C bioavailability from food versus supplements has been debated, few studies have examined how intake form affects absorption and physiological markers. Methods: This randomized, controlled, crossover trial aimed to compare the bioavailability of vitamin C consumed as a supplement, through raw fruits and vegetables, or through fruit and vegetable juice. Twelve healthy adults underwent three 1-day crossover trials, each separated by a 2-week washout. Participants consumed 101.7 mg of vitamin C via powder, raw fruits and vegetables (186.8 g), or juice (200 mL). Plasma and urinary vitamin C concentrations, urinary metabolites (^1^H NMR), and antioxidant activity (ORAC and TRAP) were assessed over 24 h. Results: All interventions elevated plasma vitamin C levels, with juice yielding the highest AUC (25.3 ± 3.2 mg/dL·h). Urinary vitamin C increased in all groups. Metabolomics revealed increased urinary excretion of mannitol, glycine, taurine, dimethylglycine (DMG), and asparagine, and decreased choline and dimethylamine (DMA). Notably, urinary mannitol increased only in the morning. Choline significantly decreased after powder intake (*p* = 0.001), with similar trends observed in the other groups. DMG and glycine increased following raw and juiced vegetable intake. Antioxidant activity showed transient ORAC elevation post-powder but no sustained improvements. Conclusions: Vitamin C is bioavailable from all intake forms, with juice providing the most efficient absorption. Urinary metabolite changes suggest microbiota-related modulation, while antioxidant activity improvements were limited.

## 1. Introduction

L-ascorbic acid (vitamin C) is an indispensable water-soluble compound that supports essential physiological processes in humans. While most animals are able to synthesize vitamin C internally, humans and a few other mammals have lost this capacity because of a mutation in the *gulonolactone oxidase* gene, which encodes the key enzyme required for vitamin C biosynthesis [1]. Consequently, vitamin C must be obtained from dietary sources. Vitamin C plays an important role in maintaining normal physiological functions. It is required for the metabolism of tyrosine, folic acid, and tryptophan and participates in hydroxylation reactions involving glycine, proline, and catecholamines [2]. It also contributes to the conversion of cholesterol to bile acids, thereby lowering serum cholesterol levels, and enhances the intestinal absorption of iron.

In addition to these roles, vitamin C serves as a strong antioxidant, scavenging reactive oxygen species (ROS) in the body [3,4]. It also regenerates other antioxidants, including vitamin E, by reducing their radical forms [5,6]. Additionally, vitamin C is indispensable for collagen biosynthesis, an essential process for wound healing [7], and it helps regulate immune responses by promoting T cell differentiation and antibody production by B cells [2].

Deficiency of vitamin C can lead to clinical manifestations such as scurvy [8], anemia, poor wound healing, muscle weakness, and increased susceptibility to infections [2]. Vitamin C absorption occurs primarily through sodium-dependent vitamin C transporters (SVCTs) [9], while its oxidized form, dehydroascorbic acid (DHA), enters cells via glucose transporters (GLUTs) and is subsequently reduced to ascorbic acid [10]. The relative contribution of DHA intake to overall vitamin C homeostasis remains unclear.

Gastrointestinal absorption of vitamin C is regulated in a dose-dependent manner [11]. At intake amounts between 30 and 180 mg/day, absorption efficiency ranges from 70 to 90%, but it declines to below 50% when intake exceeds 1 g/day, with excess being excreted through urine [5]. Pharmacokinetic data indicate that an oral dose of 1.25 g ascorbic acid produces a mean plasma concentration of approximately 135 µmol/L—roughly twice that achieved by a dietary intake of 200–300 mg from vitamin C-rich foods [10]. Even repeated doses of 3 g every 4 h do not raise plasma concentrations beyond 220 µmol/L, and repeated doses of 2 g every 4 h do not raise plasma concentrations beyond 300 mg [5]. Within tissues, millimolar concentrations are found in leukocytes, the eyes, adrenal and pituitary gland, and the brain, while extracellular fluids such as plasma and saliva contain lower micromolar levels.

Plasma vitamin C concentrations increase rapidly with daily intakes above 30 mg, plateauing at approximately 70–80 µmol/L, indicating a renal threshold beyond which urinary excretion rises proportionally. Daily intakes of 50–90 mg are linearly correlated with plasma and neutrophil levels, whereas intakes above 100 mg lead to tissue saturation. At 200 mg/day, further increases in plasma concentration are minimal, indicating that a steady state has been achieved [12,13].

Inadequate consumption of fruits and vegetables remains a public health issue. Diets rich in these foods are associated with lower risks of chronic disease, including cardiovascular disorders and cancer. The World Health Organization (WHO) recommends consuming at least 500 g of fruits and vegetables per day, while the Korean Nutrition Society recommends 210–490 g of vegetables and 300–600 g of fruits daily. However, according to the 2022 Korea National Health and Nutrition Examination Survey (KNHANES), only 22.7% of individuals aged ≥ 6 years met the recommendation, representing a decrease from 35.6% in 2013; the 2023 survey reported a further drop to 22.1%. These data indicate that a majority of the population consistently fails to achieve adequate intake, potentially leading to insufficient vitamin C consumption.

The COVID-19 pandemic has heightened global awareness of the relationship between diet and health, encouraging increased consumption of fruits and vegetables in some populations and a growing interest in fresh juices [14]. Although juice products are often perceived as being nutrient-rich, few studies have directly compared the bioavailability of vitamin C between juiced forms of fruits and vegetables. Accordingly, the current study was designed to compare the bioavailability of vitamin C obtained from raw fruits and vegetables, their juices, and vitamin C supplements using equivalent doses and to evaluate whether these different intake forms affect antioxidant activity, thereby elucidating the physiological impact of vitamin C according to intake type.

The novelty of this study lies in its integrated and translational approach. Using a randomized, crossover design with equivalent vitamin C doses, we compared supplement, raw, and juiced forms within the same individuals while controlling for background diet. By simultaneously assessing plasma kinetics, urinary metabolites, and antioxidant markers, this study provides the first comprehensive insight into how food processing and matrix composition influence short-term vitamin C bioavailability in humans. We hypothesized that mechanical processing (juicing) would enhance short-term vitamin C absorption but not necessarily antioxidant capacity.

## 2. Materials and Methods

### 2.1. Materials

Vitamin C (ascorbic acid, ≥99%, food-grade, product number A92902), deuterium oxide (D_2_O, ≥99.9 atom % D, NMR grade), 3-(trimethylsilyl)propionic-2,2,3,3-D_4_ acid sodium salt (TSP, ≥98%, NMR grade), sodium azide (NaN_3_, ≥99.5%), 2-methylpropionamidine dihydrochloride (≥98%), 2,2′-azobis(2-methylpropionamidine) dihydrochloride (AAPH, ≥97%), fluorescein sodium salt (≥97%), Trolox (≥97%, water-soluble vitamin E analog), and ABTS (2,2′-azino-bis(3-ethylbenzothiazoline-6-sulfonic acid), ≥98%) were purchased from Sigma-Aldrich (St. Louis, MO, USA). Hydrogen peroxide (H_2_O_2_, 30%, analytical grade), metmyoglobin (from equine skeletal muscle), and Histopaque 1077 were also obtained from Sigma-Aldrich.

Phosphate-buffered saline (PBS, pH 7.4), butylated hydroxytoluene (BHT, ≥99%, HPLC grade), ammonium acetate (≥98%, HPLC grade), HPLC-grade water, and ammonium formate (≥99%, HPLC grade) were used for extraction and chromatographic analysis. All solvents and reagents used for LC-MS/MS analysis were of HPLC or LC-MS grade unless otherwise specified.

### 2.2. Preparation of Equivalent Vitamin C Sources: Raw Fruits and Vegetables and Their Juice

Fresh mandarin oranges (*Citrus reticulata*, Jeju origin), cherry tomatoes (*Solanum lycopersicum*), and orange bell peppers (*Capsicum annuum*) were purchased from a domestic online grocery store in April 2023. All items were produced in Korea; the mandarin oranges were confirmed to be of Jeju origin. Because the products were commercially distributed, the exact varieties and ripeness grades could not be verified, but all samples were selected in market-ready condition, judged by typical color and firmness at the time of purchase. The edible portions of each item were thoroughly washed, separated, and frozen at −80 °C. After freezing, the samples were freeze-dried and ground into powder. The powders were stored at −80 °C until vitamin C analysis.

To prepare the fruit and vegetable juice, washed mandarin oranges, cherry tomatoes, and orange bell pepper were blended using a low-speed blender juicer (H410; Hurom Co., Ltd., Seoul, Republic of Korea) in a ratio of 1.0:0.5:1.0 (mandarin orange/cherry tomato/orange bell pepper). The juice was freshly prepared using a juicer immediately before consumption under minimal light exposure to prevent oxidation. Each serving was provided to participants within approximately 5 min of juicing, without storage or freezing, to ensure the preservation of vitamin C content. The resulting juice was immediately frozen at −80 °C, freeze-dried, and ground into powder, which was also stored at −80 °C for vitamin C analysis. 

### 2.3. Vitamin C Concentration of Raw Fruits and Vegetables and Their Juice

For vitamin C quantification, 50 mg freeze-dried samples of mandarin orange, cherry tomato, and orange bell pepper were each mixed with 700 µL of 10 mM ammonium acetate, 650 µL of 70% methanol, and 50 µL of 0.1% butylated hydroxytoluene (BHT). The mixtures were vortexed for 5 min and sonicated at room temperature for an additional 5 min. After centrifugation at 3000 rpm for 5 min, the supernatants were filtered through a PTFE syringe filter (0.45 µm). The filtered extracts were analyzed using a Thermo Scientific Dionex Ultimate 3000 UHPLC system (Thermo Fisher Scientific, Bremen, Germany) coupled with a mass spectrometer. Vitamin C was quantified as ascorbic acid only, without chemical reduction for dehydroascorbic acid, and therefore represents the reduced form of vitamin C. Component separation was performed on an Eclipse Plus C18 column (4.6 mm × 150 mm, 5 µm; Agilent, Santa Clara, CA, USA). The mobile phases consisted of HPLC-grade water (A) and 20 mM ammonium formate (B). A linear gradient was applied, increasing B from 40% to 95% in the first minute, and held at 95% for 10 min. The total run time was 10 min, with a flow rate of 0.3 mL/min, a column temperature of 25 °C, and an injection volume of 10 µL [15].

### 2.4. Subjects and Study Design

This study was designed as a randomized, controlled, crossover trial with three groups, namely A group (a vitamin C supplement with 101.7 mg of vitamin C), B group (raw fruits and vegetables (186.8 g), including mandarin orange (74.7 g), cherry tomato (37.4 g), and orange bell pepper (74.7 g)), or C group (fruit and vegetable juice (200 mL), equivalent to 202.5 g), with each group receiving an equivalent dose of 101.7 mg of vitamin C. This standardized dose was based on the measured vitamin C content of 200 mL of fresh juice, which contained 101.7 mg of vitamin C (Table 1). Because 200 mL represents a typical single serving of fruit and vegetable juices, this volume was selected as a practical reference to evaluate vitamin C absorption and bioavailability under real-world dietary conditions. Participants were recruited in April 2023 and participated in three 1-day experimental trials, with each session separated by a 2-week interval. Twelve healthy adults, both male and female, were recruited. None of the participants were smokers, and they were instructed to refrain from consuming raw or processed fruits and vegetables rich in vitamin C, as well as any foods or supplements containing vitamin C, for one week prior to the experiment. To assess the participants’ physical characteristics, measurements were taken for height, weight, BMI, waist circumference, hip circumference, blood pressure, and blood glucose levels. During the experimental period, participants were provided with standardized meals, as shown in Table 2, and these meals did not contain vitamin C. Blood samples were collected from each participant before consumption (0 h) and 0.5, 1, 2, 4, 6, 8, 12, and 24 h after consumption. Urine samples were collected at the same time points as the blood samples: 0 h (baseline, before consumption), +2 h (2–3 h post-intake), +8 h (8–9 h post-intake), and +24 h (23–24 h post-intake) after consumption. The collected blood samples were used to analyze the bioavailability of vitamin C and to assess antioxidant capacity. This study was approved by the Institutional Review Board (IRB) of Kyungnam University (approval number: KUIRB 1040460-A-2023-005), and informed consent was obtained from participants who volunteered for the study.

### 2.5. Blood and Urine Sample Collection

Blood and urine samples were collected from a total of 12 participants after obtaining written informed consent. The remaining blood was centrifuged at 1000 rpm for 15 min, and 1 mL of the supernatant from each tube was collected for the analysis of vitamin C content. To separate plasma and serum, the samples were further centrifuged at 3000 rpm for 30 min. The separated fractions were stored at −80 °C for subsequent antioxidant activity assays. Blood samples were processed into heparinized plasma and serum immediately after collection, aliquoted to minimize oxidation, and stored at −80 °C until analysis. Urine samples were collected for two different analyses: vitamin C quantification and nuclear magnetic resonance (NMR) spectroscopy. Plasma and urine samples were aliquoted and stored at −80 °C immediately after collection without the addition of any antioxidant or stabilizing agent. All urine samples were aliquoted immediately after collection and stored at −80 °C to prevent oxidation and metabolite degradation. For vitamin C analysis, urine samples were centrifuged at 1500 rpm for 10 min, and the resulting supernatant was collected and stored at −80 °C until further analysis. For NMR analysis, samples were thawed at 4 °C and centrifuged at 13,000 rpm for 10 min at the same temperature. Then, 540 μL of the supernatant was mixed with 60 μL of 0.15 M phosphate buffer (pH 7.4) prepared in deuterium oxide (D_2_O), containing 0.05% 3-(trimethylsilyl) propionic-2,2,3,3-D_4_ acid sodium salt (TSP) and 0.04% sodium azide (NaN_3_). TSP was used as a chemical shift reference at 0.00 ppm for proton detection, NaN_3_ was added to prevent microbial spoilage of the sample, and D_2_O provided a lock signal for the NMR spectrometer.

### 2.6. Quantitative Analysis of Vitamin C in Plasma and Urine

Vitamin C levels in plasma and urine were quantified using the same LC-MS/MS conditions described for the analysis of dried fruit and vegetable samples. Pre-treated plasma and urine samples were analyzed using a Thermo Scientific Dionex Ultimate 3000 UHPLC system (Thermo Fisher Scientific, Bremen, Germany). Component separation was carried out on an Eclipse Plus C18 column (4.6 mm × 150 mm, 5 µm; Agilent, USA). The mobile phases consisted of HPLC-grade water (A) and 20 mM ammonium formate (B). A linear gradient was applied to increase solvent B from 40% to 95% over 1 min, and then it was maintained at 95% for 10 min. The total run time was 10 min. The flow rate was set at 0.3 mL/min, the column temperature was maintained at 25 °C, and the injection volume was 10 µL.

### 2.7. ^1^H NMR Analysis

All one-dimensional (1D) ^1^H NMR spectra of urine were acquired using a Bruker Advance 700 spectrometer (Bruker Biospin Gmbh, Rheinstetten, Germany) equipped with a cryogenic triple-resonance probe and a Bruker automatic injector, operating at a 700.40 MHz ^1^H frequency and 298 K. Spectral data were accumulated by using a pulse sequence including presaturation of the water resonance (noesygppr1d), with an acquisition time of 1.95 s, a recycle delay of 2 s, 128 scans, and a sweep width of 8147.51 Hz, which led to 9.25 min of total acquisition for each sample. Tuning, matching, and shimming were automatically carried out on each sample during automatic acquisition. Zero-filling to 32 k points and line-broadening of 0.3 Hz were applied to each free induction decay before Fourier transformation. Phase correction of the spectra was manually carried out using Topspin (version 2.1, Bruker Biospin Gmbh). Signal assignment for representative urine sample was facilitated by two-dimensional (2D) total correlation spectroscopy (TOCSY) and heteronuclear single-quantum correlation (HSQC) experiments.

### 2.8. Plasma Antioxidant Activity

#### 2.8.1. Oxygen Radical Absorbance Capacity (ORAC) Assay

The antioxidant capacity was evaluated using a fluorescein-based ORAC assay [16]. Peroxyl radicals were generated by adding 5 mM 2,2′-azobis(2-methylpropionamidine) dihydrochloride (AAPH). The fluorescein working solution (40 nM) was prepared following the procedure described by Ou et al. [17]. Fluorescence was monitored with a FLUOstar microplate reader (BMG Labtech, Ortenberg, Germany) at excitation and emission wavelengths of 485 nm and 535 nm, respectively. The antioxidant activity was expressed as micromoles of trolox equivalents (μM TE) per gram of sample based on the area under the fluorescence decay curve corresponding to 1 μM trolox, a water-soluble analog of vitamin E (6-hydroxy-2,5,7,8-tetramethylchroman-2-carbonyl acid).

#### 2.8.2. Total Radical-Trapping Antioxidant Parameter (TRAP) Assay

The TRAP of plasma was assessed using a modified inhibition assay as previously reported by Rice-Evans and Miller [18]. This method quantifies the degree of suppression of the ABTS radical cation, produced through the reaction between ferryl myoglobin intermediates and ABTS {2,2′-azinobis (3-ethylbenzothiazoline 6-sulfonate), 150 mM} and in the presence of metmyoglobin (25 mM) with hydrogen peroxide (75 mM). The inhibition of absorbance reflects the antioxidant capacity within the plasma sample (0.84% *v*/*v*). Samples were incubated at 30 °C for 8 min, and absorbance changes were measured at 740 nm using a UV/VIS spectrophotometer (Shimadzu Corporation, Kyoto, Japan). Total antioxidant capacity was calculated using a trolox calibration curve and expressed as trolox equivalent antioxidant capacity (TEAC, mM).

### 2.9. Statistical Analysis

All data, except for NMR data, were entered using an MS Excel database system and analyzed using SPSS (version 25.0) for Windows (IBM Co., Armonk, NY, USA). Percentages and mean values ± standard error of the mean (SE) were calculated for each variable. Statistical analysis was performed using a repeated-measures one-way analysis of variance (ANOVA) to evaluate differences among interventions, with participant ID included as a random factor to account for within-subject variability inherent in the crossover design. When significant main effects were detected, post hoc pairwise comparisons were performed using the Bonferroni adjustment. A *p*-value < 0.05 was considered statistically significant. NMR spectra corrected for phase and baseline were transformed to American Standard Code for Information Interchange (ASCII) format, calibrated to proton of glucose at 5.23 ppm, and aligned using the icoshift method in MATLAB (R2010b, Mathworks Inc., Natick, MA, USA). The regions corresponding to residual water were removed prior to integral normalization or constant sum normalization. Normalization is a common method of scaling each spectrum to the same virtual concentration in order to avoid the dilution effect of metabolites in any sample set. After normalization, the resulting data set was used for multivariate statistical analysis. In particular, the orthogonal projection to latent structures (OPLS) model, a supervised pattern recognition method, was used to elicit maximum information about discriminant compounds from the sample data in which MATLAB with scripts developed at Imperial College London, United Kingdom, were used for generation of OPLS models with a color-coded correlation coefficient [19].

## 3. Results

### 3.1. Vitamin C Concentration in Raw Fruits and Vegetables and Their Juices

**Figure 1:**
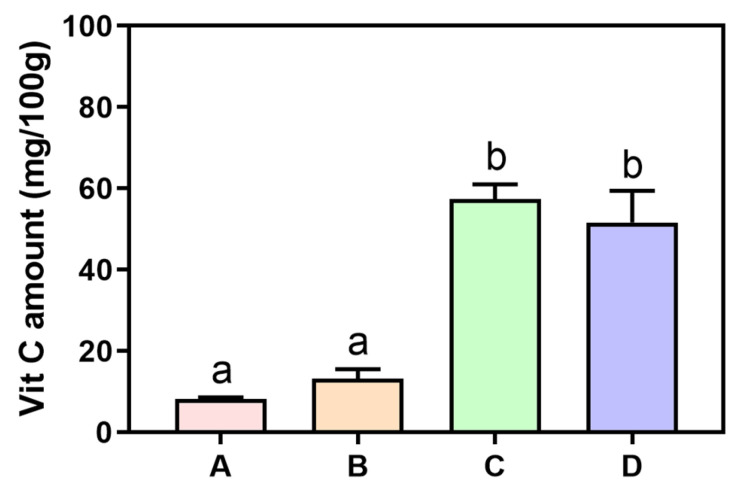
Vitamin C content of the test ingredients (mg/100 g, fresh weight). A, mandarin orange; B, cherry tomato; C, orange bell pepper; D, fruit and vegetable juice. Data are presented as mean ± SE (*n* = 3). The blended juice was prepared from mandarin orange, cherry tomato, and orange bell pepper mixed in a 1.0:0.5:1.0 ratio. Different letters above the bars indicate significant differences according to a one-way ANOVA followed by Bonferroni’s post hoc test (*p* < 0.05).

To determine the vitamin C content per 100 g of fresh mandarin orange, cherry tomato, and orange bell pepper and their juices, each sample was analyzed using ascorbic acid as a standard compound (Figure 1). The measured vitamin C content was 8.01 ± 0.38 mg/100 g for mandarin orange, 14.31 ± 1.21 mg/100 g for cherry tomato, 56.45 ± 2.48 mg/100 g for orange bell pepper, and 50.21 ± 8.90 mg/100 g for the mixed juice. Although the vitamin C contents in mandarin orange and cherry tomato were relatively low, the high vitamin C level in orange bell pepper contributed to a well-maintained overall vitamin C content in the mixed juice when blended in a 1.0:0.5:1.0 ratio. Based on these results, the vitamin C content in 200 mL of the juice used for the clinical trial was calculated to be 101.7 mg.

### 3.2. Characteristics of Subjects

The study was conducted on 12 healthy adults (six males and six females) using a randomized, controlled, crossover trial design. When comparing the baseline characteristics of the participants by group (Table 3), there were no significant differences in any of the variables, including age, sex, height, weight, BMI, blood pressure, waist circumference, and hip circumference.

### 3.3. Plasma and Urine Vitamin C Concentration

**Figure 2:**
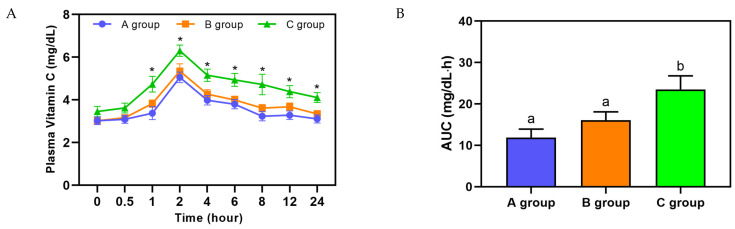
Plasma vitamin C concentration and area under the curve (AUC) following vitamin C supplement, raw fruit and vegetable, and fruit and vegetable juice intake. (**A**) Time-dependent changes in plasma vitamin C levels after vitamin C supplement, raw fruit and vegetable, and fruit and vegetable juice intake. (**B**) Area under the curve (AUC) of plasma vitamin C concentrations calculated from the time-dependent changes shown in A. A group, vitamin C supplement; B group, raw fruit and vegetable; C group, fruit and vegetable juices. Mean ± S.E. *: significantly different at *p* < 0.05 compared to the A group at the same time point according to Student’s *t*-test. ^a,b^: Different letters (a, b) above the bars indicate significant differences among groups according to Duncan’s multiple range test (*p* < 0.05).

Plasma vitamin C concentrations were measured using HPLC 0, 0.5, 1, 2, 4, 6, 8, 12, and 24 h after the intake of the vitamin C supplement (A group), raw fruits and vegetables (B group), and fruit and vegetable juice (C group). In all groups, plasma vitamin C levels began to increase at 1 h and reached peak levels 2 h post-intake. At 2, 4, 6, 8, and 12 h after intake, the C group exhibited significantly higher plasma vitamin C concentrations compared to the A and B groups (Figure 2A). The area under the curve (AUC) for plasma vitamin C concentrations further confirmed this trend, with the C group showing the highest value (23.5± 3.2 mg/dL·h), followed by the A group (11.9 ± 2.1 mg/dL·h) and B group (16.1 ± 2.0 mg/dL·h) (Figure 2B).

Urinary vitamin C concentrations were assessed to evaluate the excretion pattern following intake of the same interventions. Urine samples were collected at −2 h (baseline), +2 h (2–3 h post-intake), +8 h (8–9 h post-intake), and +24 h (23–24 h post-intake). In all groups, urinary vitamin C concentrations significantly increased from +8 h post-intake onward. However, no statistically significant differences were observed in urinary vitamin C levels among the three groups (Table 4).

### 3.4. ^1^H NMR Analysis of Participants’ Urinary Metabolites

**Figure 3:**
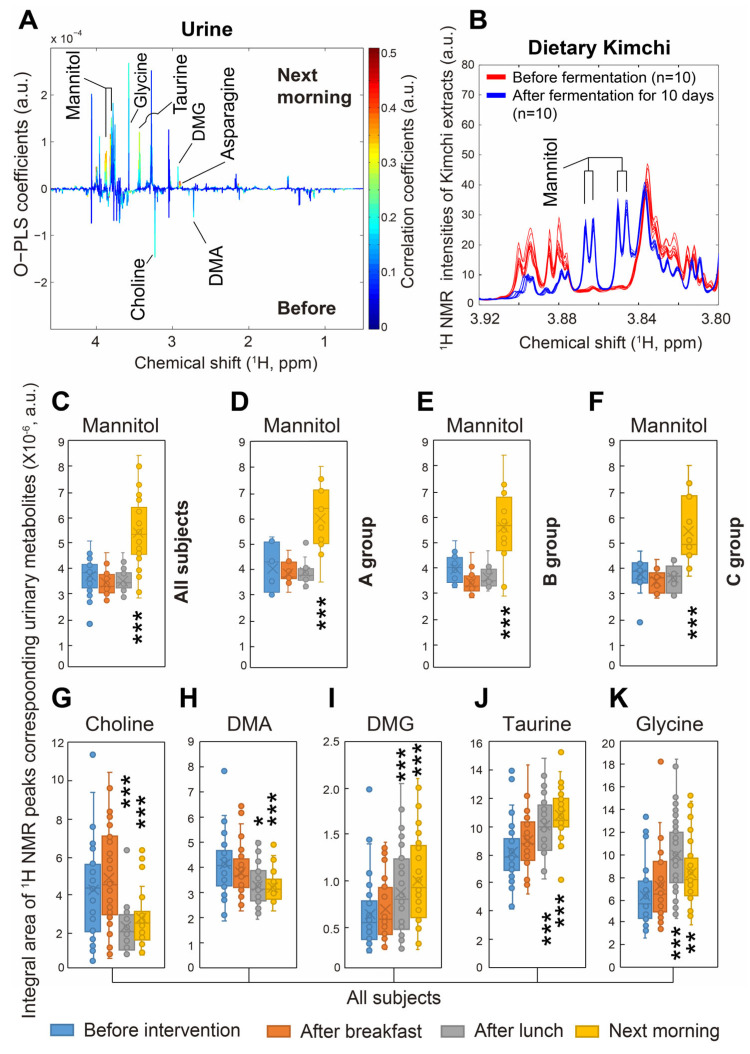
Urinary metabolite profiles and changes 24 h after consumption of vitamin C supplement, raw fruits and vegetables, and fruit and vegetable juices. Statistical total correlation spectroscopy (STOCSY) generated from partial ^1^H NMR urinary spectrum of all participants for identifications of urinary metabolites changed after dietary intervention for 24 h (*n* = 36) compared to before dietary intervention (*n* = 36) (**A**), and partial raw ^1^H NMR spectrum of dietary kimchi obtained before fermentation with lactic acid bacteria of *Leuconsotoc mesenteroides*, one of the major bacteria commonly used for kimchi fermentation (red, *n* = 10), and after fermentation for 10 days (blue, *n* = 10) (**B**). In panel (**A**), the upper section demonstrates higher amounts of metabolites in urine collected the morning following the dietary intervention compared to those collected in the morning before the dietary intervention. The relative amounts of urinary metabolites in panels (**C**–**K**) were determined by calculations of the integral area of ^1^H NMR peaks corresponding to urinary metabolites following integral normalization. DMA, dimethylamine; DMG, dimethylglycine. *, **, and *** indicate the significance of differences at the levels of *p* < 0.05, *p* < 0.01, and *p* < 0.001, respectively, in comparison with the amounts before the dietary intervention. 3-HIB, 3-hydorxyisobutyrate; 2-HIB, 2-hydorxyisobutyrate; 3-HIV, 3-hydroxyisovalerate; 4-DEA, 4-deoxyerythronic acid; 4-DTA, 4-deoxythreonic acid; NAG, *N*-acetyl glycoprotein; DMA, dimethylamine; DMG, dimethylglycine; TMAO, trimethylamine *N*-oxide; MG, methylguanidine.

^1^H NMR-based metabolites identified in urine are shown in Supplementary Figure S1. STOCSY analysis accelerated the identifications of urinary metabolites perturbed following dietary intervention, showing increased levels of urinary mannitol, glycine, taurine, dimethylglycine (DMG), and asparagine and decreased levels of urinary choline and dimethylamine (DMA) after dietary intervention with vitamin C supplement, raw fruits and vegetables, and fruit and vegetable juices for 24 h (Figure 3). It was not surprising that acute changes in urinary metabolites occurred with the 24 h dietary intervention in the present study as the influence of acute phytochemical intake on human urinary metabolomic profiles has been reported previously [20].

Increased amounts of urinary mannitol after intakes of vitamin C-rich raw vegetables and its juices, as well as vitamin C supplement, were observed in urine collected only in the morning following the dietary intervention (Figure 3C–F).

**Figure 4:**
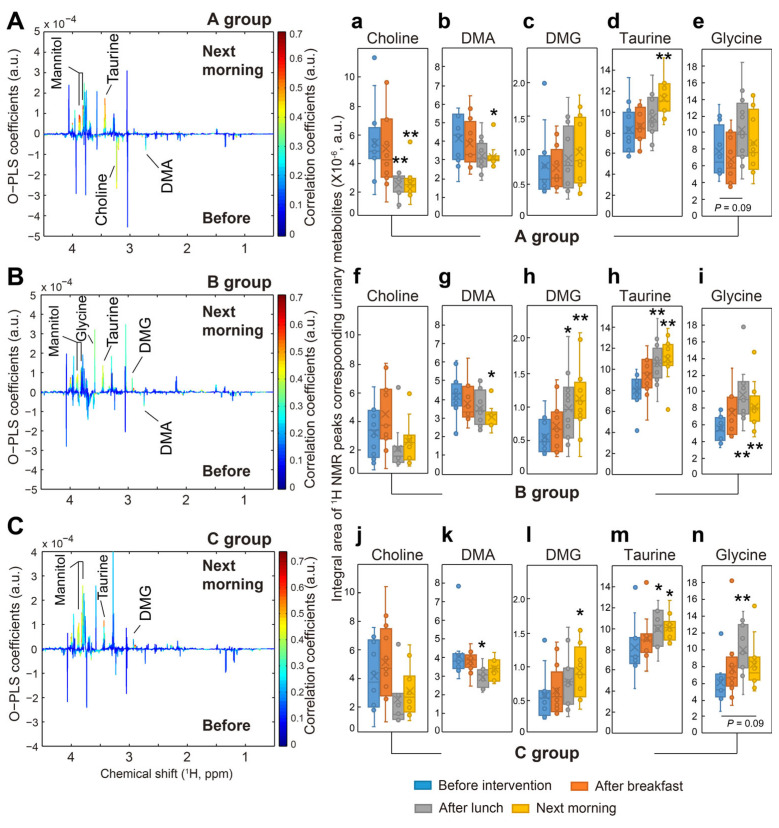
Changes in urinary metabolites and relative concentrations 24 h after consumption of vitamin C supplement, raw fruit and vegetables, and fruit and vegetable juices. Perturbations of urinary metabolites during the 24 h dietary interventions involving a vitamin C supplement ((**A**), *n* = 12), raw fruits and vegetables ((**B**), *n* = 12), and fruit and vegetable juices ((**C**), *n* = 12). Panels ‘a’ to ‘n’ show changes in relative amounts of selected urinary metabolites at different collection times in the groups who received a vitamin C supplement (**a**–**e**), raw fruits and vegetables (**f**–**i**), and fruit and vegetable juices (**j**–**n**). *, *p* < 0.05; **, *p* < 0.01. DMA, dimethylamine; DMG, dimethylglycine.

The levels of taurine in the urine of all subjects started to increase after the first dietary intervention and then significantly increased after the 24 h dietary intervention (Figure 4d,h,m). Dietary taurine possibly contributes to the increases in the levels of urinary taurine. In fact, all diets in the present study consisted of small pieces of chicken, which is food known to be rich in taurine. In particular, chicken was provided for a late-night snack to all subjects during the three interventions. However, taurine levels in the urine collected before dinner and the next morning were not significantly increased in each dietary intervention group.

Urinary choline levels started to decrease after the second dietary intervention with the vitamin C supplement, raw fruits and vegetables, and fruit and vegetable juices in the present study, even though the decreases were only statistically significant in the subjects who received vitamin C powder supplementation (*p* = 0.001). But the amounts of urinary choline showed a strong decreasing trend in the subjects who consumed raw fruits and vegetables and fruit and vegetable juices at the level of *p* = 0.100 (Figure 4). TMA and TMAO were also identified in the urine of all subjects in the present study, but their levels were not changed. Moreover, the levels of urinary dimethylglycine (DMG) were significantly increased in subjects who consumed raw fruits and vegetables or fruit and vegetable juice following the second dietary intervention, but they showed an increasing trend in subjects with vitamin C supplement, maybe due to large variations among the subjects.

### 3.5. Plasma Antioxidant Activity

**ORAC.** Plasma antioxidant activity, as measured by ORAC values, was assessed 0, 0.5, 1, 2, 4, 6, 8, 12, and 24 h after the intake of the vitamin C supplement (A group), raw fruits and vegetables (B group), and fruit and vegetable juices (C group) (Table 5). At 0.5 h post-intake, the A group showed significantly higher plasma ORAC values compared to the B and C groups. However, this increase was transient, and no significant differences were observed among the groups at later time points.

**TRAP.** Plasma total antioxidant capacity was measured 0, 0.5, 1, 2, 4, 6, 8, 12, and 24 h after the intake of the vitamin C supplement (A group), raw fruits and vegetables (B group), and fruit and vegetable juices (C group) (Table 5). No significant differences were observed over time within each group, and there were no significant differences among the groups at any time point.

## 4. Discussion

This study provides an integrated comparison of equivalent vitamin C doses from supplements, raw fruits and vegetables, and blended juices in a randomized, controlled, crossover trial involving 12 healthy adults. Participants consumed either a vitamin C supplement, raw fruits and vegetables, or fruit and vegetable juice in a single dose, and their vitamin C absorption, urinary metabolites, and antioxidant effects were evaluated. Plasma vitamin C concentrations peaked 2 h post-consumption in all groups, with significantly higher concentrations observed in the fruit and vegetable juice group. Additionally, the area under the curve (AUC) for plasma vitamin C was the greatest in the juice group, suggesting that the bioavailability of vitamin C is not compromised when consumed in juice form compared to whole fruits and vegetables.

Our findings are consistent with previous studies indicating that vitamin C is rapidly absorbed within 1 to 3 h post-intake and returns to baseline within 24 h. Graumlich et al. [11] also demonstrated a rapid rise in plasma vitamin C concentrations following supplementation, followed by a decline to baseline levels within 24 h. Furthermore, a study by Vinson and Bose [21] reported that vitamin C consumed with a natural citrus extract containing flavonoids, proteins, and carbohydrates showed approximately 35% greater bioavailability—based on plasma concentrations—than synthetic ascorbic acid alone. However, a randomized crossover study comparing synthetic vitamin C tablets and natural kiwifruit-derived vitamin C showed similar absorption patterns, with plasma concentrations peaking between 0.5 and 1 h and no significant differences between the two forms [22]. Likewise, Carr and Vissers [23] reviewed ten clinical studies comparing the absorption of vitamin C alone versus in flavonoid-rich foods and found no significant differences in bioavailability.

Our integrated approach allowed for simultaneous assessment of plasma kinetics, urinary metabolites, and antioxidant capacity, providing a comprehensive understanding of how food processing and matrix composition influence short-term vitamin C bioavailability in humans. Collectively, these findings suggest that although food matrix components such as flavonoids or macronutrients may slightly affect absorption kinetics, the overall bioavailability of vitamin C remains comparable across supplement, whole food, and juice forms.

Regarding the compositions of diets provided during the dietary intervention in the current study, all subjects were given kimchi stew only for dinner during the three interventions. We compared the amounts of mannitol, which is commonly known to be produced during kimchi fermentation with *lactic acid bacteria* (LAB) of *Leuconostoc mesenteroides* [24], and found that mannitol was produced in large amounts in kimchi (Figure 3B). Therefore, the increased amounts of urinary mannitol observed in the morning after the 24 h dietary intervention might have been caused by the diet with mannitol-rich kimchi stew in the current study. Nevertheless, since mannitol is a precursor of butyrate and propionate and known to be a healthy beneficial compound in the gut lumen, kimchi consumption might be a potential reason for butyrate and propionate production in the gut [25]. However, fecal metabolite composition was not explored in the present study. Moreover, we believed that mannitol in kimchi stew did not affect urinary choline, dimethylamine (DMA), dimethylglycine (DMG), taurine, or glycine in all groups (Figure 3 and Figure 4), demonstrating that these urinary metabolites were changed by vitamin C supplementation.

Taurine is a sulfur-containing amino acid and an important signaling molecule in the intestine. It is well known that taurine regulates immune responses and enhances immunity. For example, taurine supplementation significantly regulated intestinal microflora and boosted intestinal immunity under gut microbiota dysbiosis in mice, which resulted in the reconstitution of intestinal flora homeostasis [26]. Daily supplementation of vitamin C showed microbiota-modulating effects in healthy individuals, with several beneficial shifts in bacterial populations [27,28]. Therefore, it was suggested that increased urinary taurine in all subjects undergoing dietary interventions in the present study might be a results of the modulation of gut microbiota by vitamin C supplement, raw fruit and vegetable, or fruit and vegetable juice intake.

Dietary choline is metabolized to trimethylamine (TMA) in the gut by intestinal microbiota and then converted into trimethylamine *N*-oxide (TMAO) by hepatic oxidation through flavin monooxygenase 3 in the liver. Significant amounts of circulating TMAO cause atherosclerosis and increase risks for cardiovascular disease and cardiac events, such as myocardial infarction and stroke [29,30]. This metabolic status of urinary DMG was very similar to that of urinary glycine, likely because glycine is synthesized from DMG and sarcosine [31]. Indeed, vitamin C-modulated microbiota may lead to increased urinary glycine through deconjugation of glycine-bound BAs [32]. Therefore, dietary intervention with vitamin C supplement or vegetables rich in vitamin C might lead to the conversion of dietary choline to DMG and then glycine through modulations of gut microbial compositions, avoiding its metabolism to TMA, a precursor of TMAO. However, the associations between gut modulation and vitamin C supplements remain to be further investigated.

In parallel, this study also examined the effects of these dietary interventions on antioxidant capacity in plasma. Following the intake of vitamin C supplement, raw fruits and vegetables, or fruit and vegetable juices, plasma vitamin C concentrations increased rapidly and peaked 2 h post-intake. Among antioxidant markers, ORAC values exhibited a transient but significant increase after intake and were then maintained over time. However, TRAP values showed no significant changes over time or between groups. These findings suggest that while vitamin C intake may enhance certain aspects of peroxyl radical scavenging measured by ORAC, it may not significantly affect the broader spectrum of antioxidant defenses represented by TRAP.

## 5. Conclusions

In conclusion, this randomized, controlled, crossover trial demonstrates that vitamin C from supplements, raw fruits and vegetables, or fruit and vegetable juices is efficiently absorbed, with peak plasma concentrations observed 2 h post-intake. Importantly, the juice group exhibited the highest plasma vitamin C levels and area under the curve (AUC), suggesting comparable or slightly enhanced bioavailability relative to the whole food form. Metabolomic analysis revealed favorable shifts in urinary biomarkers, such as increased excretion of glycine, taurine, and mannitol, potentially reflecting vitamin C-induced modulation of gut microbiota and associated metabolic pathways. While plasma antioxidant capacity, as measured by ORAC, showed a transient improvement after intake, TRAP values remained unchanged across groups. Collectively, these findings highlight the physiological significance of vitamin C intake, particularly when consumed in juice form, in promoting short-term bioavailability, influencing microbially associated metabolites, and contributing to antioxidant defense mechanisms.

## Figures and Tables

**Table 1:** Vitamin C concentrations in vitamin C supplements, raw fruits and vegetables, and fruit and vegetable juices.

	Amount	Vitamin C Content
Vitamin C supplements	101.7 mg	101.7 mg
Raw fruits and vegetables		
Tangerine	74.7 g	101.7 mg
Cherry tomato	37.4 g	
Orange bell pepper	74.7 g	
Fruit and vegetable juices	202.5 g (200 mL)	101.7 mg

**Table 2:** Nutritional composition of standardized meals provided to participants during the experimental period.

	Menu
Meal types	
Lunch	Cooked rice Fried egg Grilled chicken drumsticks (boneless thigh meat) Seasoned dried shredded fish (Ilmi muchim) Seasoned dried filefish strips Fried pollock skin Braised quail eggs
Afternoon snacks	Steamed white rice cake Energy bar
Dinner	Cooked rice Aged kimchi stew with thinly sliced beef brisket Korean-style rolled omelet grilled chicken wings Braised peanuts Seasoned dried shredded fish (Ilmi muchim) Seasoned dried filefish strips Fried pollock skin Braised quail eggs
Evening snacks	Grilled chicken Carbonated water
	**Nutrient contents**
Energy	
Calorie (kcal)	3363.2
Carbohydrate (g)	329.0
Protein (g)	318.6
Fats (g)	120.3
Vitamin	
Vitamin C (mg)	0.0

**Table 3:** Subjects’ characteristics.

	A Group (*n* = 12) ^(1)^	B Group (*n* = 12)	C Group (*n* = 12)
**Sex**			
Male (*n*)	6	6	6
Female (*n*)	6	6	6
**Age (years)**	22.0 ± 0.5 ^(3), ns(4)^	22.0 ± 0.5	21.8 ± 0.7
**Hight (kg)**	167.5 ± 1.9 ^ns^	167.3 ± 1.8	167.91 ± 1.5
**Weight (kg)**	78.5 ± 6.7 ^ns^	78.9 ± 6.8	72.45 ± 5.4
**BMI (kg/m^2^)** ^(2)^	27.8 ± 2.2 ^ns^	28.0 ± 2.2	25.55 ± 1.7
**SBP (mmHg)**	130.3 ± 6.1 ^ns^	127.8 ± 3.9	123.83 ± 3.7
**DBP (mmHg)**	80.6 ± 4.9 ^ns^	75.3 ± 3.1	73.67 ± 3.3
**WC (cm)**	90.8 ± 5.0 ^ns^	91.5 ± 5.0	83.72 ± 4.1
**HC (cm)**	103.6 ± 3.6 ^ns^	103.3 ± 3.6	100.75 ± 3.4

^(1)^ A group, vitamin C supplement; B group, raw fruits and vegetables; C group, fruit and vegetable juices. ^(2)^ BMI, body mass index; SBP, systolic blood pressure; DBP, diastolic blood pressure. ^(3)^ Mean ± S.E. ^(4)^ No significant differences (*p* < 0.05) between time points within each group according to one-way ANOVA followed by Duncan’s multiple range test.

**Table 4:** Effects of vitamin C supplement, raw fruit and vegetable, and fruit and vegetable juice intake on urine vitamin C concentration.

Time	A Group (*n* = 12) ^(1)^	B Group (*n* = 12)	C Group (*n* = 12)
−2 h	1.41 ± 0.16 ^(2), a(3), NS(4)^	1.08 ± 0.06 ^a^	0.96 ± 0.05 ^a^
+2 h	0.92 ± 0.05 ^a, NS^	1.01 ± 0.06 ^a^	0.94 ± 0.06 ^a^
+8 h	0.91 ± 0.05 ^b, NS^	0.98 ± 0.08 ^b^	1.04 ± 0.05 ^b^
+24 h	0.98 ± 0.07 ^c, NS^	1.00 ± 0.05 ^c^	1.07 ± 0.06 ^c^

^(1)^ A group, vitamin C supplement; B group, raw fruit and vegetable; C group, fruit and vegetable juices. ^(2)^ Mean ± S.E. ^(3)^ a–c; values with different letters are significantly different at *p* < 0.05 after Duncan’s multiple range test with time. ^(4)^ NS, no significant difference (*p* < 0.05) between groups at the same time point according to one-way ANOVA followed by Duncan’s multiple range test.

**Table 5:** Plasma antioxidant activity (ORAC and TRAP) following vitamin C supplement, raw fruit and vegetable, and fruit and vegetable juices intake.

	A Group (*n* = 12) ^(1)^	B Group (*n* = 12)	C Group (*n* = 12)
**ORAC (μM TE)** ^(2)^			
0 h	342.0 ± 33.2 ^(3), a(4), ns(5)^	308.8 ± 23.2 ^a^	290.9 ± 16.1 ^a^
0.5 h	465.2 ± 29.1 ^bc, B(6)^	339.7 ± 34.0 ^ab, A^	343.6 ± 31.7 ^ab, A^
1 h	407.8 ± 35.7 ^abc, ns^	452.9 ± 45.9 ^c^	416.2 ± 33.2 ^bc^
2 h	399.9 ± 23.1 ^abc, ns^	481.9 ± 32.9 ^c^	412.8 ± 48.6 ^bc^
4 h	380.4 ± 33.0 ^ab, ns^	429.8 ± 34.7 ^bc^	395.0 ± 33.5 ^bc^
6 h	453.2 ± 28.8 ^bc, ns^	489.7 ± 26.0 ^c^	446.6 ± 29.6 ^bc^
8 h	469.5 ± 20.4 ^bc, ns^	468.7 ± 53.0 ^c^	447.9 ± 36.9 ^bc^
12 h	479.1 ± 22.6 ^c, ns^	504.4 ± 22.6 ^c^	423.9 ± 39.0 ^bc^
24 h	460.0 ± 34.3 ^bc, ns^	435.7 ± 23.5 ^bc^	459.4 ± 37.4 ^c^
**TRAP (mM)**			
0 h	1.04 ± 0.05 ^NS (7), ns^	1.08 ± 0.06 ^ns^	0.96 ± 0.05 ^ns^
0.5 h	0.95 ± 0.06 ^NS^	1.02 ± 0.06	0.98 ± 0.07
1 h	0.97 ± 0.06 ^NS^	1.05 ± 0.04	1.03 ± 0.07
2 h	1.00 ± 0.06 ^NS^	0.99 ± 0.06	1.03 ± 0.07
4 h	0.89 ± 0.06 ^NS^	0.98 ± 0.06	0.97 ± 0.07
6 h	0.98 ± 0.06 ^NS^	1.08 ± 0.05	1.04 ± 0.03
8 h	0.92 ± 0.05 ^NS^	1.01 ± 0.06	0.94 ± 0.06
12 h	0.91 ± 0.05 ^NS^	0.98 ± 0.08	1.04 ± 0.05
24 h	0.98 ± 0.07 ^NS^	1.00 ± 0.05	1.07 ± 0.06

^(1)^ A group, vitamin C supplement; B group, raw fruit and vegetables; C group, fruit and vegetable juices. ^(2)^ ORAC, Oxygen Radical Absorbance Capacity; TRAP, total radical-trapping antioxidant potential. ^(3)^ Mean ± S.E. ^(4)^ a–c; values with different letters are significantly different (*p* < 0.05) over time according to Duncan’s multiple range test. ^(5)^ ns, no significant difference (*p* < 0.05) between groups at the same time point according to one-way ANOVA followed by Duncan’s multiple range test. ^(6)^ A,B; values with different letters are significantly different at *p* < 0.05 after Duncan’s multiple range test between groups at the same time point. ^(7)^ NS, no significant difference (*p* > 0.05) within the same group over time according to one-way ANOVA followed by Duncan’s multiple range test.

## Data Availability

The data presented in this study are available upon reasonable request to the corresponding author. The data are not publicly available due to privacy restrictions.

## Supplementary Materials

The following supporting information can be downloaded at: https://www.mdpi.com/article/10.3390/nu17213331/s1, Figure S1: Urinary metabolite profile on partial representative 700 MHz 1H NMR spectrum of human urine.
