# Supplementary material for: Comparative Bioavailability of Vitamin C After Short-Term Consumption of Raw Fruits and Vegetables and Their Juices: A Randomized Crossover Study
Source: Nutrients. 2025 Oct 23;17(21):3331. doi: 10.3390/nu17213331 (PMC12608462; doi:10.3390/nu17213331)
Supplement: Supplementary file 1 [file nutrients-17-03331-s001.zip › nutrients-3938143-supplementary.pdf]

Supplementary Information

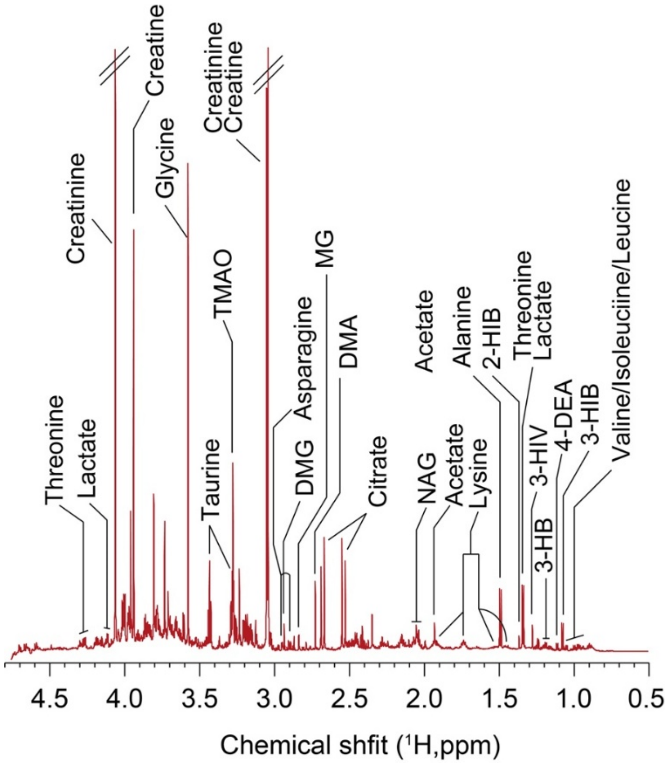

Figure S1. Urinary metabolite profile on partial representative 700 MHz 1H NMR spectrum of human urine. DMA, dimethylamine; DMG, dimethylglycine; MG, methylguanidine; TMAO, trimethylamine N-oxide; NAG, N-acetyl glycoprotein; 3-HIB, 3-hydroxyisobutyrate; 2-HIB, 2-hydroxyisobutyrate; 3-HIV, 3-hydroxyisovalerate; 4-DTA, 4-deoxythreonic acid; 4-DEA, 4-deoxyerythronic acid.
